# Treatment of medication-related osteonecrosis of the jaw with cell therapy

**DOI:** 10.3389/fcell.2024.1338376

**Published:** 2024-01-26

**Authors:** Cyril Lucien Bouland, Rokneddine Javadian, Sarah Gilis, Antoine Yanni, Maxime Le Clercq, Pierre Mestrallet, Stelianos Kampouridis, Dominique Bron, Martin Lalmand, Xavier Vanden Eynden, Edward Boutremans, Didier Dequanter, Isabelle Loeb, Pierre Philippart, Laurence Lagneaux, Nathalie Meuleman

**Affiliations:** ^1^ Laboratory of Clinical Cell Therapy (LCCT), Jules Bordet Institute, Université Libre de Bruxelles (ULB), Brussels, Belgium; ^2^ Department of Maxillofacial Surgery, Saint-Pierre Hospital, Université Libre de Bruxelles (ULB), Brussels, Belgium; ^3^ Department of Maxillofacial Surgery, EpiCURA, Hornu, Belgium; ^4^ Department of Radiology, Saint-Pierre Hospital, Université Libre de Bruxelles (ULB), Brussels, Belgium; ^5^ Department of Haematology, Jules Bordet Institute, Université Libre de Bruxelles (ULB), Brussels, Belgium

**Keywords:** medication-related osteonecrosis of the jaw (MRONJ), MRONJ, stromal vascular fraction (SVF), SVF, leukocyte-platelet-rich fibrin, L-PRF, cell therapy

## Abstract

**Introduction:** Medication-related osteonecrosis of the jaw (MRONJ) poses a significant challenge considering the absence of a “gold standard” treatment. Cell-based therapy and tissue engineering offer promising therapeutic alternatives. This study aimed to harness the regenerative properties of adipose-tissue stromal vascular fraction (AT-SVF) and leukocyte-platelet-rich fibrin (L-PRF) for MRONJ treatment. AT-SVF contains mesenchymal stromal cells (MSC) and endothelial progenitor cells (EPC), which promote bone formation, while the L-PRF scaffold can serve as a three-dimensional scaffold for the AT-SVF and support tissue healing through growth factor release.

**Materials and methods:** The protocol involved applying autologous AT-SVF within an L-PRF matrix following surgical debridement. Age, gender, body mass index, comorbidities, underlying oncological condition, prescribed antiresorptive treatment: BP or DMB, antiresorptive treatment duration, antiresorptive treatment potential discontinuation, number of MRONJ lesion, MRONJ location, MRONJ stage, MRONJ trigger factor were assessed for each patient. Patients underwent the procedure and were monitored for a minimum of 6 months based on clinical, biological and medical imaging criteria.

**Results:** Nine patients, with a total of ten MRONJ lesions, participated in the study. Six patients were female, and three were male, with a mean age of 68 ± 8 years. Four patients had multiple myeloma (MM), three had metastatic breast cancer, and two had metastatic prostate cancer. Seven MRONJ cases were classified as stage II, and three were classified as stage III. Soft tissue completely healed within a month after treatment in nine cases, with no clinical improvement observed in the remaining case. During follow-up, no sign of MRONJ recurrence was observed. Tridimensional medical imaging revealed bone healing 6 months after the surgical procedure. Immunophenotyping confirmed the presence of MSC and EPC in the AT-SVF: 12,6 ± 4,5% CD31^+^, 20.5 ± 7,8% CD34^+^, 34,4 ± 7,3% CD146^+^ and 54,6 ± 7,4% CD45^+^.

**Conclusion:** This prospective study introduces a potential new treatment approach for MRONJ using autologous AT-SVF within an L-PRF scaffold. Our results are encouraging and suggest the need for further investigation with a larger patient cohort to better understand the underlying mechanisms.

## 1 Introduction

Medication-related osteonecrosis of the jaw (MRONJ), first described by Marx in 2003, is characterized as an adverse drug reaction leading to progressive bone destruction in the jaw, significantly affecting a patient’s quality of life (Marx, 2003; Campisi et al., 2020; Ruggiero et al., 2022). According to the American Association of Oral and Maxillofacial Surgeons (AAOMS), MRONJ is defined by the following criteria: the presence of current or prior antiresorptive treatment, either alone or in combination with immune modulators or antiangiogenic medications; exposed bone in the maxillofacial region, or bone that can be probed through intra- or extraoral fistulas, persisting for more than 8 weeks; and no history of radiation therapy or metastatic disease affecting the jaws (Ruggiero et al., 2022). The risk of MRONJ among oncological patients is generally lower than 5%, with a range of 0%–18% for zoledronate and 0%–6.9% for denosumab (DMB) (Ruggiero et al., 2022). For osteoporotic patients, the MRONJ risk varies between 0.02%–0.05% with exposure to bisphosphonates (BP) and 0.04%–0.3% with exposure to DMB. The pathophysiology of MRONJ appears to be multifactorial, with several hypotheses proposed, including inhibition of bone remodelling (Buranaphatthana et al., 2021), inhibition of angiogenesis (Li et al., 2022), inflammation (Zhang et al., 2021), infection (Otto et al., 2021a), immune dysfunction (Zhang et al., 2021; Roato et al., 2023), and genetic factors (Guo et al., 2020). MRONJ is classified into stages ranging from stage 0 to stage III based on the disease progression. Currently, there is no accepted “gold standard” treatment, and care relies on scientific consensus and empirical approaches (Fortunato et al., 2020). Different therapies are recommended depending on the MRONJ stages defined by the AAOMS. They consist of preventive measures and conservative treatment for the early stages and invasive treatments for the later stages (Yarom et al., 2019). However, this pattern is increasingly being questioned. The surgical procedure seems to offer better results at all stages taken together. Its goal is to reduce procedure aggressiveness and to improve long-term disease control (Fusco et al., 2020).

**FIGURE 1:**
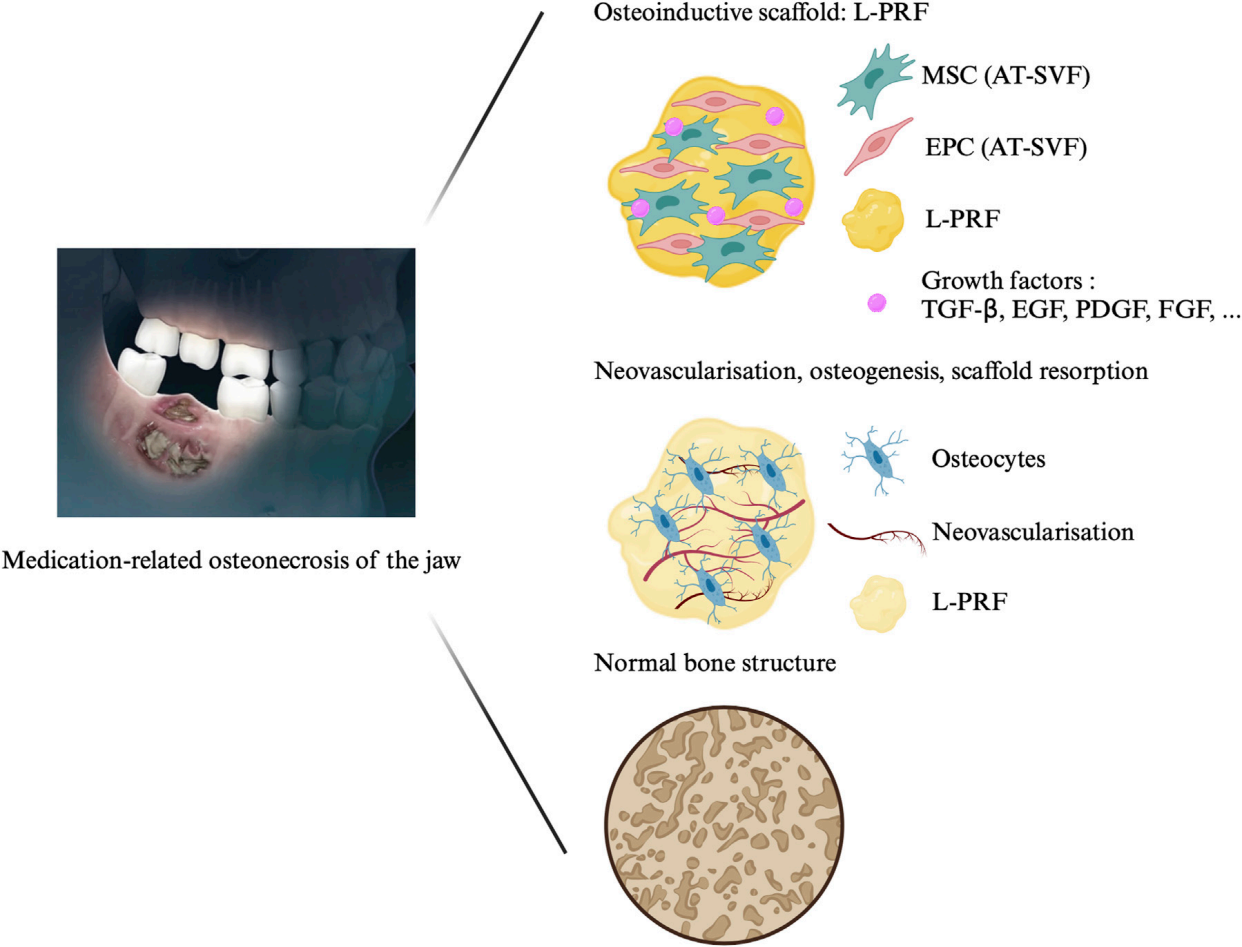
Treatment of medication-related osteonecrosis of the jaw with cell therapy. The protocol consists in the application of the association of L-PRF and AT-SVF on the surgically cleaned necrotic area. The L-PRF is obtained after centrifugation of autologous blood for 400 g during 12 min without the addition of any other components. The AT-SVF is injected in the L-PRF membrane and the association is affixed on the cleaned bone. Abbreviations: MRONJ: medication-related osteonecrosis of the jaw; L-PRF: leukocyte-platelet-rich fibrin, SVF: stromal vascular fraction, EPC: endothelial progenitor cells, AT-MSC: adipose tissue mesenchymal stromal cell; TGF-β: transforming growth factor-beta; FGF: fibroblast growth factor; PDGF: platelet-derived growth factor; EGF: Epidermal growth factor.

Several adjuvant therapies have been explored, including ozone therapy, hyperbaric oxygen (HBO), autofluorescence-guided bone surgery, pentoxifylline and tocopherol, human amniotic membrane (hAM) and autologous platelet concentrates (APC), with varying degrees of success (Fortunato et al., 2020; Govaerts et al., 2020; Odet et al., 2022). Cell therapy has also emerged as a potential treatment modality for MRONJ, supported by significant results from animal models and clinical studies (Kaibuchi et al., 2022). Recently, we published a case report on the treatment of two MRONJ patients using a combination of leukocyte-platelet-rich fibrin (L-PRF) and adipose-tissue stromal vascular fraction (AT-SVF) (Figure 1) (Bouland et al., 2021a). Bone formation requires four key elements: a cell source, a scaffold, tissue-inducing factors (signalling factors), and mechanical stimulation (Perez et al., 2018). AT-SVF, a heterogeneous cell population obtained from adipose tissue, contains mesenchymal stromal cells (MSC) and endothelial progenitor cells (EPC), among others. Through direct and indirect cellular communication, MSC and EPC promote both osteogenesis and angiogenesis, facilitating bone regeneration (Bouland et al., 2021b). L-PRF, a second-generation APC obtained by centrifugation of autologous blood without the addition of anticoagulants, could serve as a three-dimensional scaffold for the AT-SVF. Furthermore, it releases growth factors encouraging tissue healing over at least 7 days (Dohan Ehrenfest et al., 2018). This study aims to use the healing properties of AT-SVF and L-PRF to treat MRONJ.

## 2 Materials and methods

This study adhered to the Declaration of Helsinki regarding medical protocols and ethics. Approval was obtained from the Ethics Committee of Saint Pierre Hospital and EPICURA (B076201941648). All MRONJ patients provided written informed consent before participating in the study. Before undergoing the surgical procedure, patients underwent several medical examinations, including an orthopantomogram (OPG), tridimensional imaging, and a preoperative anaesthetic consultation. All cellular analyses were conducted at the Laboratory of Clinical Cell Therapy (LCCT-ULB721) of the Jules-Bordet Institute (JBI)—Université Libre de Bruxelles (ULB).

### 2.1 Characterization of the patients and the MRONJ lesions

The following factors were assessed: age, gender, body mass index (BMI) of the patients, the comorbidities, the underlying oncological condition, the prescribed antiresorptive treatment, the duration of the antiresorptive treatment, the potential discontinuation of the antiresorptive treatment, the number of MRONJ lesion per patient, the location of MRONJ, the stage of MRONJ according to the AAOMS (Ruggiero et al., 2022), the MRONJ trigger factor. These data were retrieved from the patient medical records.

### 2.2 Surgery and follow-up

**FIGURE 2:**
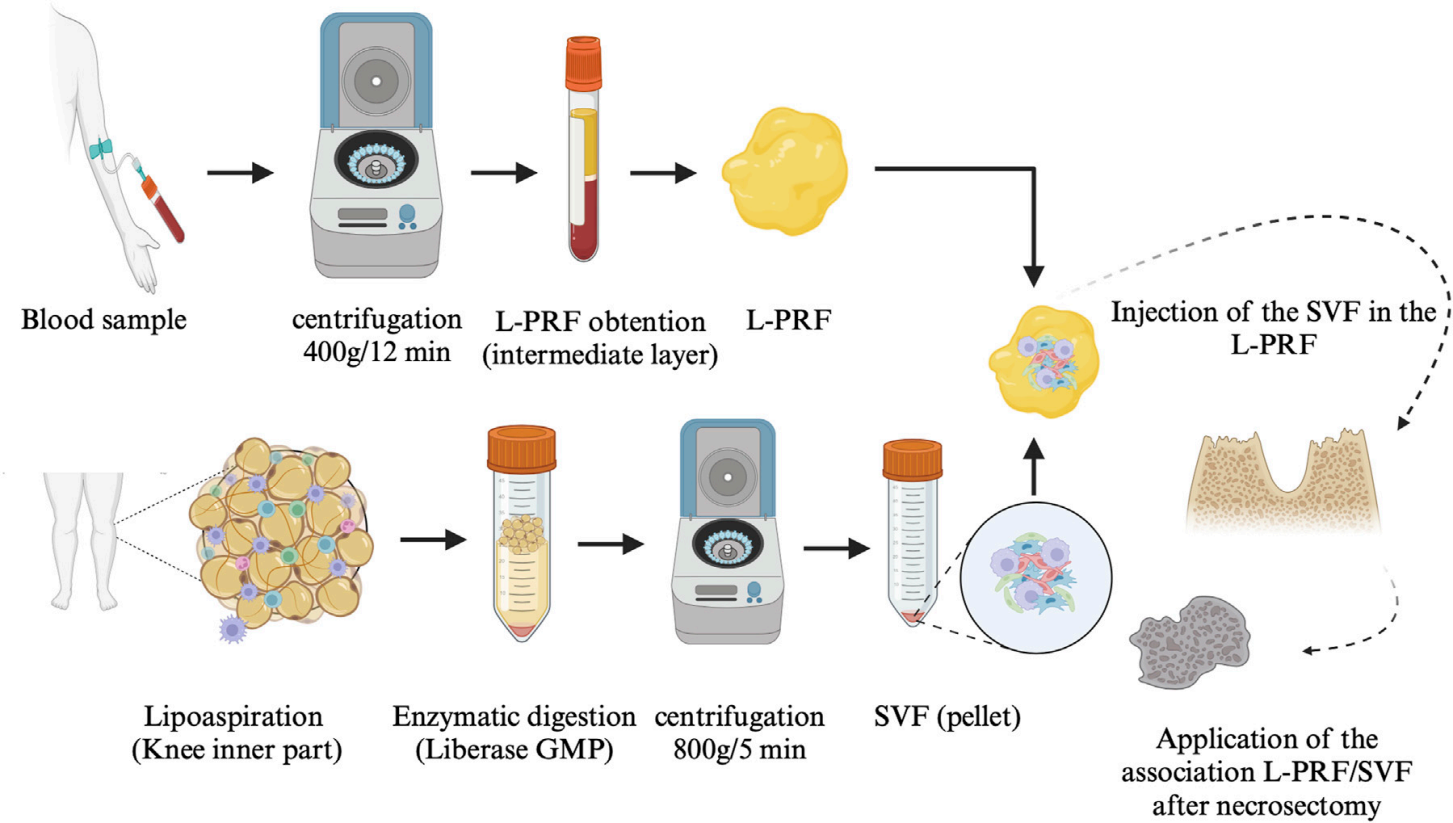
AT-SVF/L-PRF protocol Abbreviation: L-PRF: leukocyte-platelet-rich fibrin, SVF: stromal vascular fraction; GMP: good manufacturing practices.

The surgical procedure was conducted under general anaesthesia in a one-step process (Figure 2). No local anaesthesia was administered at the surgical site before the operation to avoid potential effects on local MSC proliferation (Roato et al., 2016). The procedure included AT harvest and enzymatic treatment to obtain SVF, a blood sample to prepare L-PRF, surgical debridement of the infected area based on preoperative imaging and bone vitality determined by bleeding. The procedure concluded with the application of the L-PRF scaffold containing uncultured SVF and closure of the site with a mucoperiosteal flap. Granulation tissue and necrotic bone specimens were collected for anatomopathological analysis. Postoperatively, patients received painkillers and amoxicillin/clavulanic acid 875 mg 3 times a day for 1 week. During the follow-up, patients were seen weekly for the first month, followed by monthly visits for 6 months. Three-dimensional imaging (CT scanner or CBCT) was scheduled 6 months postoperatively to assess potential bone formation.

### 2.3 Autologous AT sample and SVF preparation

A 30 mL sample of autologous AT was collected from the inner knee by lipoaspiration using a standard 3 mm multihole infusion cannula. Fat was gently aspirated using mechanical means without the use of vasoconstrictors to prevent cell cytotoxicity from local anaesthesia. The collected fat was later digested using Liberase MTF C/T, GMP grade (Creative Enzymes, Shirley, NY, United States of America) for 30 min at room temperature and then centrifuged at 800 *g* for 5 min. The resulting SVF pellet was harvested and washed with saline solution. A part of the SVF was characterized at the LCCT (cell number and immunophenotype), while the remainder was reserved for the patient’s treatment (Figure 2).

### 2.4 L-PRF preparation

A blood sample was collected via peripheral puncture using 9-mL glass collection tubes without the addition of any anticoagulants. The blood was immediately centrifuged (Intra-Spin EBA 200, Intra-Lock System, FL, United States of America) for 12 min at 400 g (2700 rpm) to obtain L-PRF (Figure 2) (Dohan Ehrenfest et al., 2018).

### 2.5 Preparation of the L-PRF scaffold containing the SVF

**FIGURE 3:**
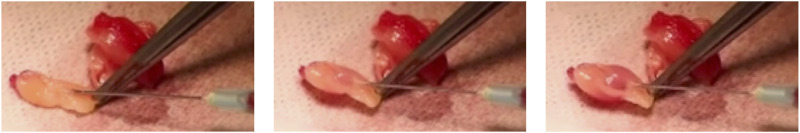
Injection of the AT-SVF in the L-PRF membrane.

The SVF was injected into the L-PRF using a microfine insulin syringe with a 23G needle, taking advantage of its three-dimensional properties (Figure 3).

### 2.6 SVF characterization

Viability and cell number in each AT-SVF were evaluated by trypan blue (Corning, Manassas, United States of America) exclusion assay. The distribution of SVF cell subpopulations was analysed by flow cytometry for cellular expression of CD31, CD34, CD146 and CD45. This allowed us to determine the respective percentages of haematopoietic (CD45^+^), stromal (CD31^-^/CD34^+^/CD146^+^/CD45^-^), and endothelial progenitors (CD31^+^/CD34^+^/CD146^+^CD45). Cell phenotyping was immediately evaluated after SVF harvest. The cells were incubated with appropriate fluorescent monoclonal antibodies for 30 min at room temperature in the dark (CD31-FITC and CD34-PE, Miltenyi Biotec, Bergisch Gladbach, Germany; CD146-PC5 and CD45-PC7, BD Pharmingen, Erembodegem, Belgium). The remaining erythrocytes were lysed, and the cells were fixed with a Uti-Lyse kit (Dako, Carpinteria, CA, United States of America) according to the manufacturer’s instructions. Data acquisition was performed on a MACSQuant analyser (Miltenyi Biotec MACS), and analysis was conducted using FCS Express 4 software (DeNovo Software).

### 2.7 Statistics

Statistical analyses were performed using GraphPad Prism version 8.3.0 for Windows (GraphPad Software, www.graphpad.com). Descriptive statistics included mean ± standard error of the mean (SEM). The evaluations consisted of clinical and radiographic analysis before and 6 months after the operation.

## 3 Results

### 3.1 Characterization of the MRONJ patients

Nine patients were included in the protocol (Table 1). Six patients were female, and three were male, with a mean age of 68 ± 8 years. Patients presented a mean BMI of 22.94 ± 0.7. All patients had an underlying oncological condition. Four patients were suffering from a multiple myeloma (MM), three a metastatic breast cancer, and two had metastatic prostate cancer. To treat those affections, antiresorptived treatments were given. DMB and BP were prescribed to four and five patients respectively. BP treatment duration lasted on average 25 ± 6 months and DMB treatment lasted on average 33 ± 7 months. In all cases except one, the treatment had been temporarily stopped after discussion with their oncologist after MRONJ onset.

**TABLE 1:** Characterization of the patients suffering from MRONJ.

Patients	Sex (F/M)	Age	BMI	Comorbidities	Oncological disease	Antiresorptive treatment	Duration of the antiresorptive treatment (months)	Discontinuation of the antiresorptive treatment (Yes/No)
Patient 1	F	68	24.21	Paroxistic arythmia	MM	BP	21	Yes
Patient 2	F	71	24.56	—	Breast cancer	BP	36	Yes
Patient 3	F	79	22.66	Haemorrhoids	Breast cancer	DMB	36	Yes
Patient 4	M	77	23.77	Inguinal hernia	MM	BP	43	Yes
				Total hip replacement				
				Phlebitis				
Patient 5	F	57	23.59	Type II diabetes	Breast cancer	DMB	46	No
				Hypertension				
Patient 6	M	61	17.96	Heart failure	Prostate cancer	DMB	15	Yes
				Hypertension				
				Appendicectomy				
Patient 7	F	72	24.35	Renal failure stage 4	MM	BP	11	Yes
				Hypertension				
				Hysterectomy				
				Ovariectomy				
Patient 8	F	59	21.36	Hypertension	MM	BP	12	Yes
Patient 9	M	73	24.03	Hypertension	Prostate cancer	DMB	36	Yes
				COPD				
				Coronary bypass				
				Colon cancer				

Abbreviations: BMI: body mass index; BP: bisphosphonate; DMB: denosumab; F: female; M: male; MM: multiple myeloma; MRONJ: medication-related osteonecrosis of the jaw. Antiresorptive treatment: Bisphosphonate or Denosumab.

### 3.2 Characterization of the MRONJ lesions

In total, ten lesions were treated with this protocol (Table 2). Eight patients had a single MRONJ lesion, while one patient had two lesions. MRONJ was located in the maxilla in three cases, the mandible in five cases, and both jaws in one case. Four patients developed MRONJ after a tooth extraction, one after a dental implant placement, one after a chronic tooth infection. No trigger factor was found for the last three patients.

**TABLE 2:** Characterization of MRONJ.

Patients	MRONJ number	MRONJ location	MRONJ stage	MRONJ trigger factor
Patient 1	1	Mandible	II	Chronic tooth infection
Patient 2	1	Mandible	II	Dental implant placement
Patient 3	1	Mandible	II	Tooth extraction
Patient 4	2	Mandible and Maxilla	II	Tooth extraction
Patient 5	1	Mandible	II	Tooth extraction
Patient 6	1	Mandible	II	Tooth extraction
Patient 7	1	Maxilla	III	—
Patient 8	1	Maxilla	III	—
Patient 9	1	Maxilla	III	—

Abbreviations: MRONJ: medication-related osteonecrosis of the jaw.

### 3.3 AT-SVF/L-PRF protocol

**FIGURE 4:**
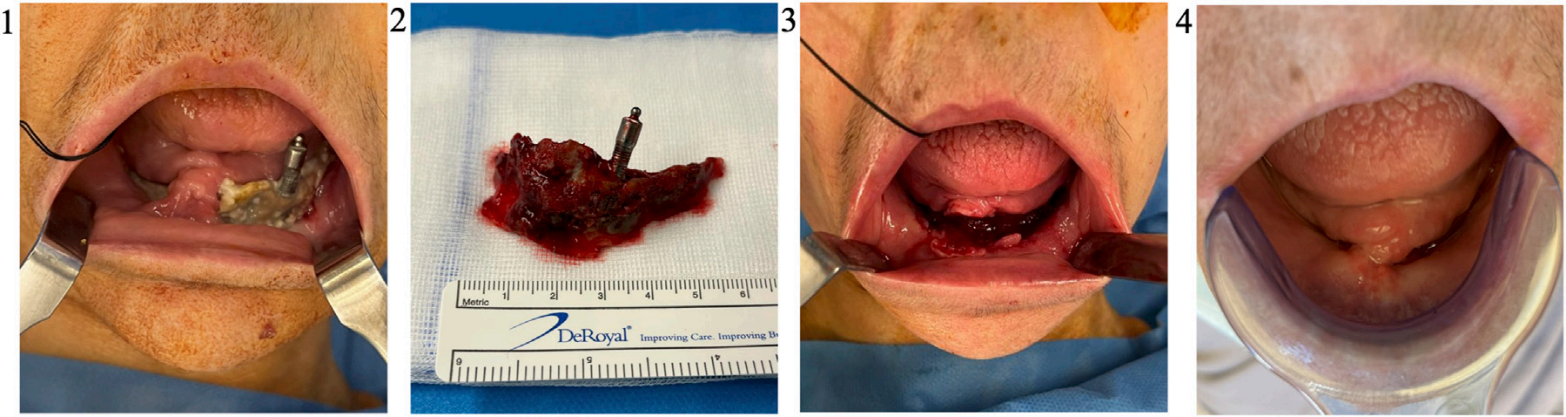
Necrosectomy and follow-up (Patient 2). Patient 2 was, a 71 years old female, suffering from a metastatic breast cancer. She presented a stage II MRONJ bisphosphonate intake for 36 months. She developed MRONJ after a dental implant placement. Before the procedure, she presented a large bone sequestrum located in the symphysis of the mandible (1). After a sequestrectomy, the necrotic bone (2) is sent in the anatomopathology department for analysis. The MRONJ site is surgically cleaned (3) before the application of the AT-SVF/L-PRF. One month after the procedure, a complete soft tissue healing was highlighted (4). Abbreviations: MRONJ: medication-related osteonecrosis of the jaw; L-PRF: leukocyte-platelet-rich fibrin, AT-SVF: adipose tissue stromal vascular fraction.

**FIGURE 5:**
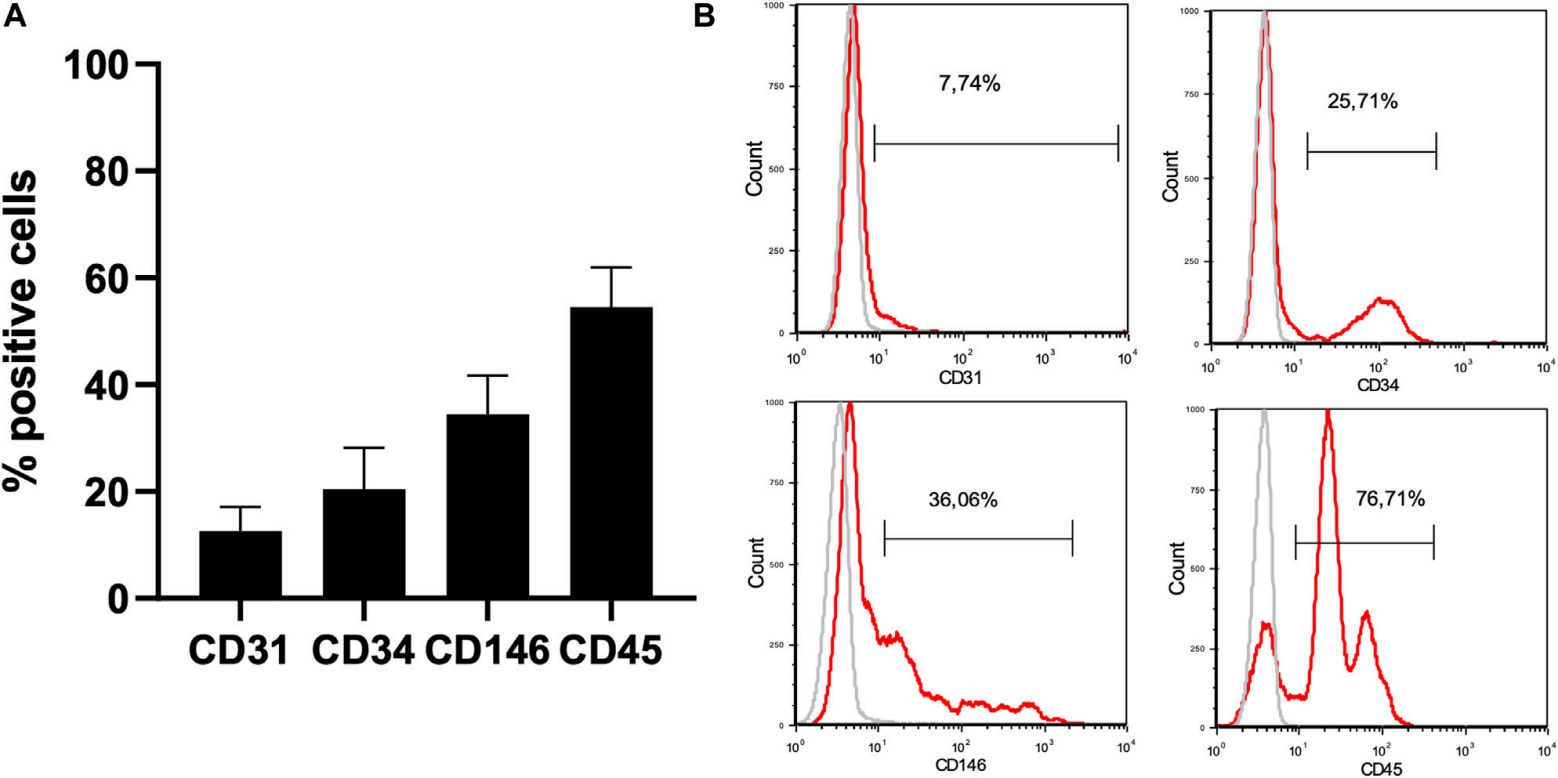
Characterization of the AT-SVF by flow cytometry. 1. Phenotypic characterization of the AT-SVF (N = 9) by flow cytometry: CD31^+^: 12,6 ± 4,5% CD34^+^: 20.5 ± 7,8% CD146^+^: 34,4 ± 7,3% CD45^+^: 54,6 ± 7,4%. 2. Clinical and medical imaging evaluation of the protocol. Abbreviations: AT-SVF: adipose-tissue stromal vascular fraction.

All nine patients benefited from the protocol under general anaesthesia in one-day clinic (Table 3). No adverse reactions were assessed during the study. On average, 2.96 × 10^6^ ± 1.1 × 10^6^ viable cells were harvested by lipoaspiration from the inner part of the knee. A total of 0.372 × 10^6^ ± 0.08 × 10^6^ cells (0.2 mL) were injected into the three L-PRF scaffolds. Each patient benefited from a single intervention comprising resection of the necrotic bone (Figure 4) and one single application of the association L-PRF and AT-SVF. SVF was obtained through AT harvesting and analysed by flow cytometry, confirming the presence of a diverse cell population containing endothelial cells, EPC, MSC, and cells from the haematopoietic lineage: 12,6 ± 4,5% CD31^+^, 20.5 ± 7,8% CD34^+^, 34,4 ± 7,3% CD146^+^ and 54,6 ± 7,4% CD45^+^. (Figure 5).

**TABLE 3:** AT-SVF/L-PRF therapy and follow-up.

Patients	Number of cells harvested/mL	Number of cells injected	Number of L-PRF membrane affixed	Anatomopathology	Results	Medical imaging	Follow-up (months)
Patient 1	3.2 × 10^5^	1 × 10^5^	3	Osteonecrosis with actinomycetes colonisation	Complete healing	Bone healing	37
				2 × 0.5 × 0.5 cm			
Patient 2	2.8 × 10^6^	5.6 × 10^5^	3	Osteonecrosis with actinomycetes colonisation	Complete healing	Bone healing	16
				6 × 2 × 0.5 cm			
Patient 3	1 × 10^6^	2 × 10^5^	3	Osteonecrosis with actinomycetes colonisation	Complete healing	Bone healing	13
Patient 4	1.44 × 10^6^	2.88 × 10^5^	Lesions 1: 3	Lesion 1: Osteonecrosis with actinomycetes colonisation	Complete healing	Bone healing	22
			Lesion 2: 3	1.5 × 1 × 0.5 cm			
				Lesion 2: Osteonecrosis with actinomycetes colonisation			
				0.5 × 0.2 cm			
Patient 5	6 × 10^5^	1.2 × 10^5^	3	Osteonecrosis with actinomycetes colonisation	No healing	Progression of the bone necrosis	19
				0.5 × 0.4 × 0.1 cm			
Patient 6	2.2 × 10^6^	4.4 × 10^5^	3	Osteonecrosis with actinomycetes colonisation	Complete healing	Statu quo	6
				5 × 3 × 0.5 cm			
Patient 7	11.2 × 10^6^	2.24 × 10^6^	3	Osteonecrosis with actinomycetes colonisation	Complete healing	Bone healing	12
				2 × 1.2 × 1.2 cm			
Patient 8	3.6 × 10^6^	7.2 × 10^5^	3	Osteonecrosis with actinomycetes colonisation	Complete healing	Bone Healing	7
				1 × 0.8 × 0.6 cm			
Patient 9	3.5 × 10^6^	7 × 10^5^	3	Osteonecrosis with actinomycetes colonisation	Complete healing	Bone healing	7
				2 × 1.5 × 1cm			

Abbreviations: cm: centimeter; L-PRF: leukocyte-platelet-rich fibrin; mL: milliliter.

### 3.4 Clinical and medical imaging evaluation of the protocol

**FIGURE 6:**
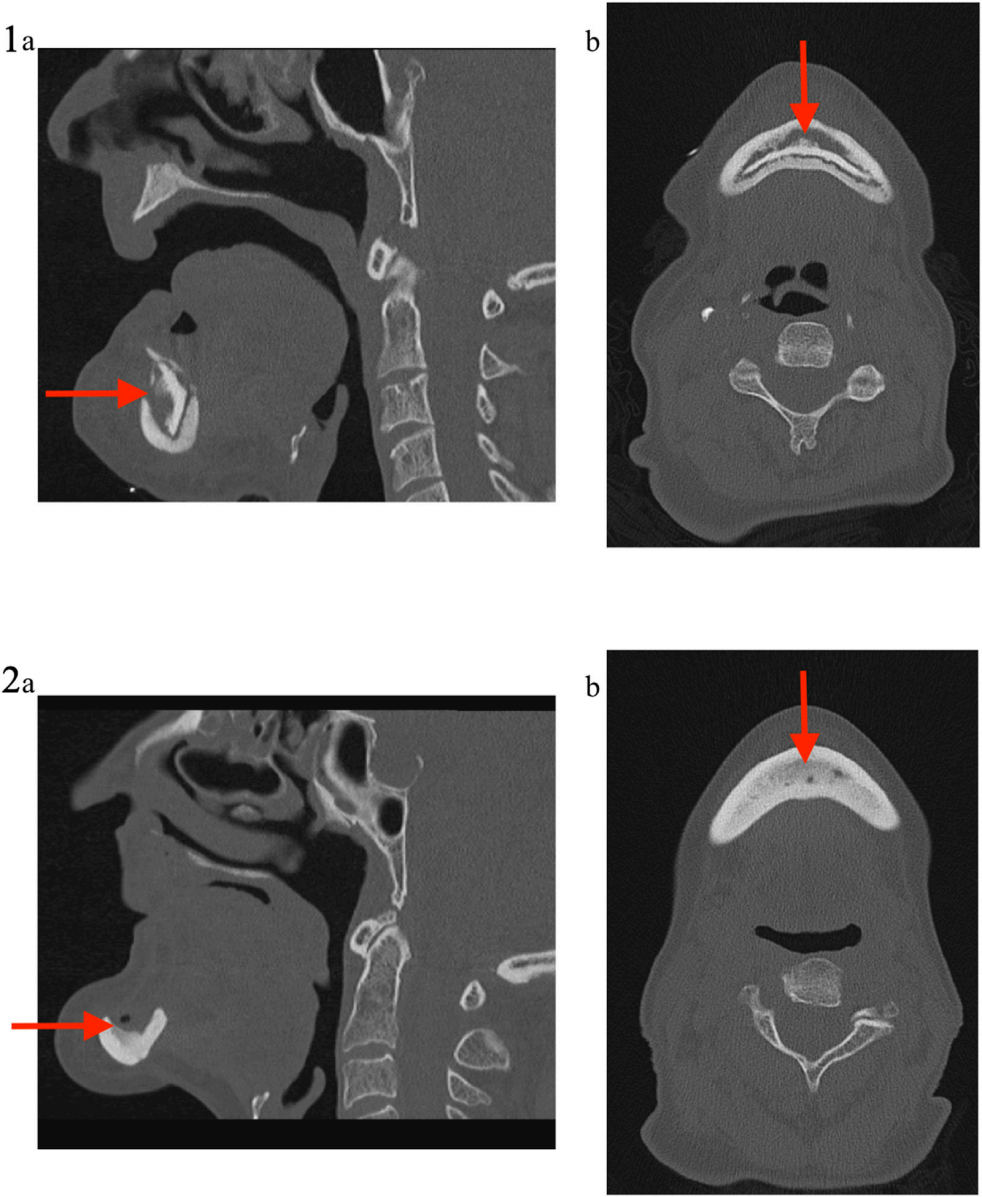
Medical imaging (Patient 2). Patient 2 was a 71 years old female, suffering from a metastatic breast cancer. She presented a stage II MRONJ after bisphosphonate intake for 36 months. She developed MRONJ after a dental implant placement. Before the procedure, she presented a large bone sequestrum located in the symphysis of the mandible. The preoperative medical imaging highlights the large bone sequestrum (1); **(A)** sagittal view and **(B)** transverse view. Six months after the intervention, the patient performed a second medical imaging displaying cortical bone rethickening, necrotic areas cleansing and increased bone density with reconstruction of the mandibular symphysis (2); **(A)** sagittal view and **(B)** transverse view.

**FIGURE 7:**
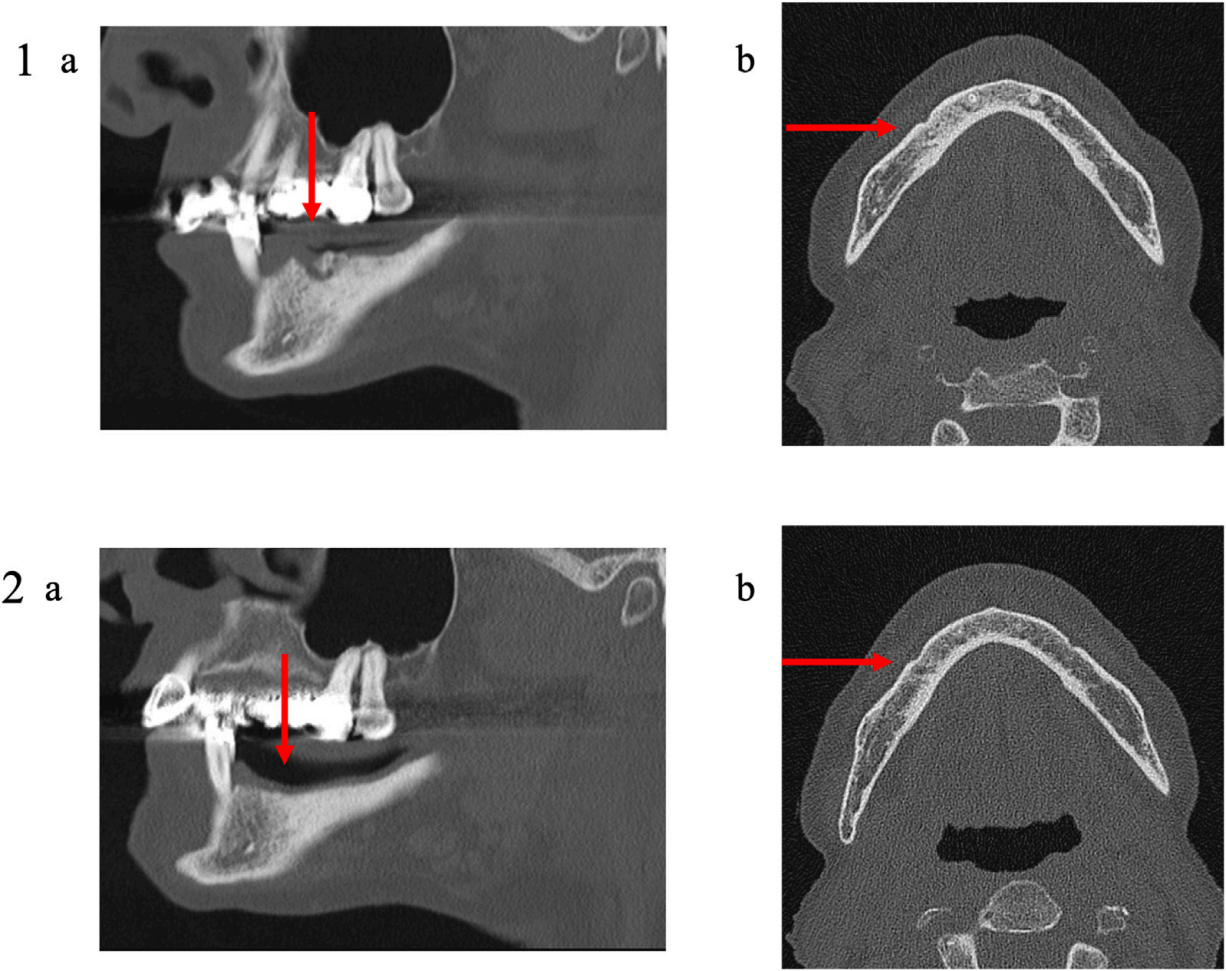
Medical imaging (Patient 3). Patient 3 was a 79 years old female suffering from a metastatic breast cancer. She presented a stage II MRONJ after denosumab intake for 36 months. She developed MRONJ after a tooth extraction. Before the procedure, she presented a large bone sequestrum located in the right parasymphysis of the mandible. The preoperative medical imaging highlights the necrotic bone and a residual apex lingual to the mental foramen (1); **(A)** oblique sagittal view and **(B)** transverse view. Six months after the intervention, the patient the patient performed a second medical imaging displaying no residual apex (avulsed during the procedure), a reduction of the trabecular densification and thinning of the lingual and the vestibular cortical bone (2); **(A)** oblique sagittal view and **(B)** transverse view.

**FIGURE 8:**
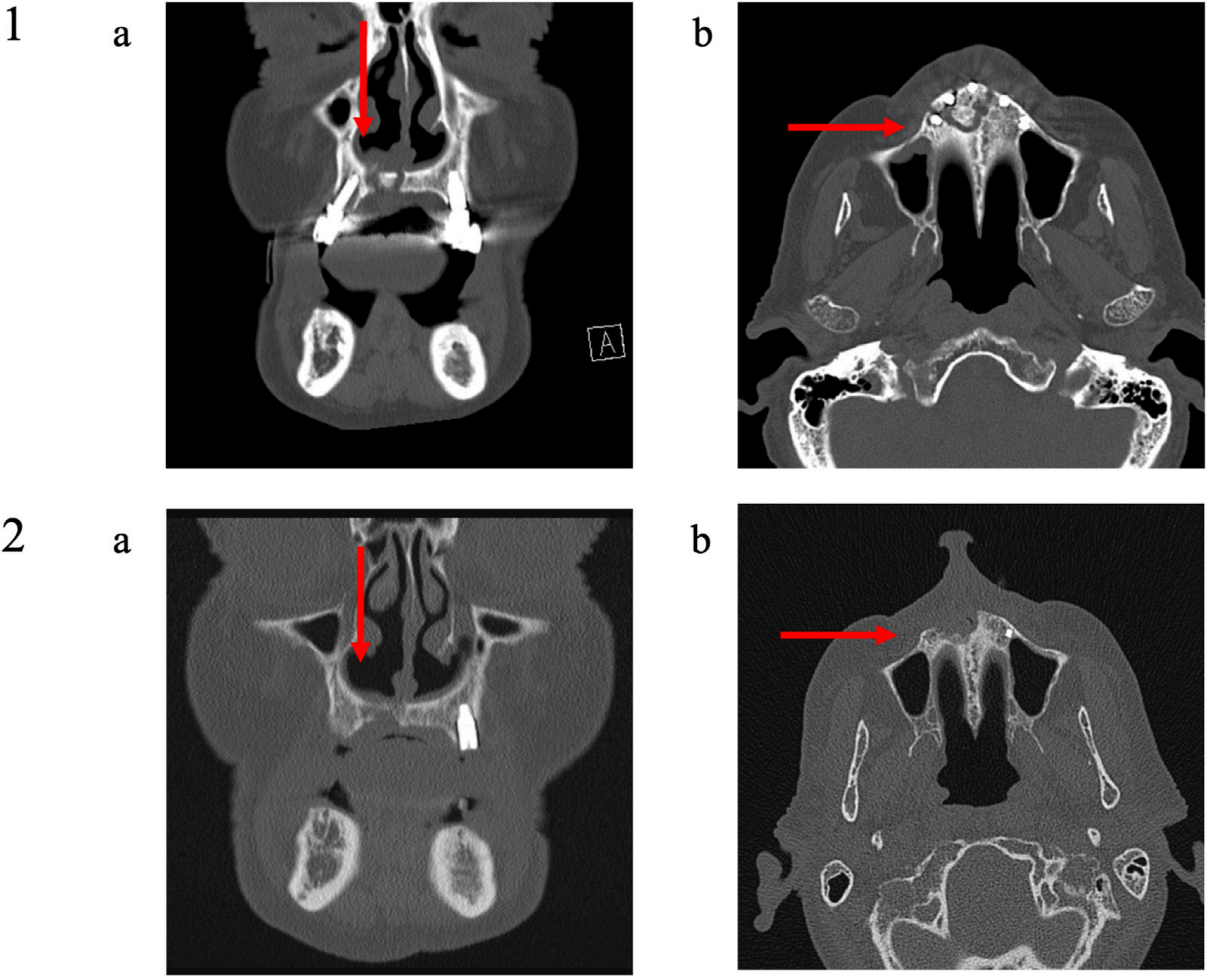
Medical imaging (Patient 9). Patient 9 was a 79 years old male suffering from a metastatic prostatic cancer. He presented a stage III MRONJ after denosumab intake for 36 months. He developed MRONJ spontaneously. Before the procedure, he presented a large bone sequestrum located in the premaxilla with an oral-nasal communication. The preoperative medical imaging highlights the necrotic bone and five dental implants (1); **(A)** frontal view and **(B)** transverse view. Six months after the intervention, the patient performed a second medical imaging displaying one residual dental implant in position 23 (The other four dental implants were removed during the procedure), a slow reconstruction of the bone tissue at the position of the dental implants 13 and 21 and at the level of the nasal floor (2); **(A)** frontal view and **(B)** transverse view.

Complete healing of the oral mucosa was observed within 1 month for nine cases. All the symptoms were resumed in the two first weeks in nine cases. Patients’ quality of life was significantly improved. The last case remained unchanged. Fortunately, the patient with no MRONJ improvement rejected the bony sequestrum naturally after 12 months. The follow-up period ranged from 6 to 37 months, with a mean follow-up of 15.44 months. No recurrence was observed during this period. Unfortunately, one patient died from cancer after 6 months of follow-up. Two patients developed new MRONJ lesions (stage II lesions) in different jaw locations and are currently undergoing treatment. After 6 months of follow-up, three-dimensional medical imaging was performed, demonstrating bone healing in eight cases and one case remained in *status quo* (Figure 6). After 6 months of follow-up, three-dimensional medical imaging was performed, demonstrating bone healing in eight cases and one case remained in status quo (Figures 6–8)

## 4 Discussion

In the current study, we investigated a potential new treatment for MRONJ using cell therapy with the combination of L-PRF and AT-SVF. The outcomes were highly encouraging. In nine MRONJ lesions out of ten, the oral mucosa healed completely within just 1 month after treatment, significantly improving the patients’ oral health and overall quality of life. No recurrence of MRONJ was observed during the follow-up period. Tridimensional medical imaging revealed evidence of bone healing, confirming the positive impact of the L-PRF/AT-SVF protocol on tissue regeneration and repair. These results align with our case report published in 2021 (Bouland et al., 2021a), where two MRONJ patients successfully benefited from the same protocol. In total, 11 patients with 12 MRONJ lesions were treated using the L-PRF/AT-SVF combination, further supporting the potential of this innovative approach. However, the contribution of each element could not be assessed: the surgery itself, AT-SVF and L-PRF. This protocol offers a potential new treatment consisting of the association of the three above-cited elements that could not be separated from one another. Furthermore, a randomized control study evaluating each element should be performed to evaluate the exact contribution of the surgery itself, AT-SVF and L-PRF.

The current medical literature lacks a consensus on treatments for MRONJ (Fortunato et al., 2020). Treatment approaches often vary based on the MRONJ stages defined by the AAOMS. Recommendations include preventive measures and conservative treatments for early-stage cases, while more invasive interventions are reserved for advanced stages (Yarom et al., 2019). However, this treatment paradigm is being increasingly questioned. Surgical procedures have shown better results, offering potential benefits across all stages of MRONJ. The primary objective of surgery is to minimize procedure aggressiveness and enhance long-term disease control, as emphasized by Fusco et al., in 2020 (Fusco et al., 2020). Studies conducted by Ristow et al. (Ristow et al., 2019) and Kaibuchi et al. (2021) have contributed valuable insights. Ristow et al. followed 75 patients with 92 lesions for an average of 16.1 ± 14.1 months (Ristow et al., 2019). Their findings indicated that conservative nonsurgical treatments rarely led to the healing of MRONJ. Of the 92 lesions, only eight healed with conservative nonsurgical approaches. In contrast, 67 worsened, and surgical intervention was eventually needed for 57 lesions. Kaibuchi et al. followed 129 patients with 129 lesions for 120 months (Kaibuchi et al., 2021). Their results showed that the overall cure rates after 12, 36, and 60 months were 25.8%, 50.8%, and 72.4%, respectively. Interestingly, the cure rate for stage I MRONJ was lower than that of stages II and III after 80 months of conservative treatment. The authors concluded that MRONJ carries a poor prognosis when treated conventionally, particularly when conservative treatment is attempted for mild cases. Ristow and others suggested that conservative management might be suitable for patients unwilling to undergo surgery or those with reduced overall health who are not suitable candidates for surgical procedures to preserve symptoms. However, the authors stressed the importance of early and consistent surgical interventions throughout all stages of the disease to prevent silent disease progression and the risk of extensive bone loss (Ristow et al., 2019).

Several adjuvant therapies have been tested with varying success rates (Govaerts et al., 2020). Freiberger considered that HBO could be helpful in Bisphosphonate-related osteonecrosis of the jaw (BRONJ) treatment. (Freiberger, 2009; Freiberger et al., 2012). HBO produces reactive oxygen and nitrogen species that modulate positively the redox-sensitive intracellular signalling molecules involved in bone turnover, counteracting the negative effect of BP on bone turnover through the disruption of osteoclast signal transduction, maturation, and longevity (Freiberger, 2009). Significantly improved healing with a reduced mean time was highlighted after HBO addition to conventional treatment. Recently, Colapinto et al. reported that pentoxyphyline and tocopherol improved healing after surgery (Colapinto et al., 2023). Ozone (O_3_) therapy, applied topically (Agrillo et al., 2012) or infiltrated (Di Fede et al., 2022), has also been suggested in combination with conventional MRONJ treatment and has shown positive results. Indeed, O_3_ plays a role in the treatment of chronic, nonhealing, or ischaemic wounds due to its antimicrobial, antioxidant and biostimulatory properties (Di Fede et al., 2022). Autofluorescence-guided bone surgery might be a tool to optimize the complete removal of necrotic bone parts and is a reliable, minimally invasive, and promising treatment option for patients with MRONJ (Pautke et al., 2011; Otto et al., 2021b). Pautke et al. and Otto et al. observed 85%–90.2% success after autofluorescence-guided bone surgery (Pautke et al., 2011; Otto et al., 2021a). Recently, Odet et al. used human amniotic membranes in combination with necrosectomy to treat MRONJ with interesting results. Improved healing was noticed in 80% of the cases. (Odet et al., 2022). APC, and especially PRF, have also been tested to treat MRONJ (Fortunato et al., 2020). However, the results in the literature are inconsistent. Fortunato et al. did not highlight any advantages to the addition of PRF in the treatment of MRONJ (Fortunato et al., 2020). However, the heterogeneity of the PRF protocols studied might be a significant cofounding factor (Miron et al., 2019). Yalcin-Ülker et al. and Zelinka et al. recently treated 19 patients with 20 MRONJ lesions and 40 patients with 40 MRONJ lesions, respectively (Zelinka et al., 2021; Yalcin-Ülker et al., 2023). After debridement or sequestrectomy and adjunction of PRF, complete resolution was observed in 80% and 85% of the cases, respectively.

These last few years, cell therapy has been investigated in MRONJ treatment (Kaibuchi et al., 2022). Several protocols have been tested in different animal models to understand the pathogenesis (Kikuiri et al., 2010; Li et al., 2013). Li et al. established a BRONJ animal model in minipigs (Li et al., 2013). BM-MSC biological and immunological properties are impaired in BP-treated minipigs. The Foxp3-positive regulatory T (Treg) cells ratio in peripheral blood is decreased, and interleukin (IL)-17 is increased in BP-treated minipig serum. After allogeneic BMMSC transplantation via intravenous infusion, mucosal healing and bone reconstruction were observed, IL-17 levels were reduced, and Tregs were elevated. Kikuiri et al. also demonstrated in a mouse model that a systemic MSC infusion prevents and cures BRONJ-like disease possibly through peripheral tolerance induction, marked by an inhibition of Th17 and an increase in Treg cells (Kikuiri et al., 2010). Ogata et al. evaluated the therapeutic effects of conditioned media (CM) from human MSC in a rat BRONJ-like model (Ogata et al., 2015). Interestingly, the antiapoptotic and anti-inflammatory effects of MSC-CM drastically regulate local bone turnover. Significant socket healing was observed after MSC-CM intravenous injection. Histological analysis showed new bone formation and the appearance of osteoclasts in the MSC-CM group. In parallel, Barba-Recreo and others observed that adipose-derived stem cell (ASC)-based treatments seem to prevent MRONJ more effectively than mucosal flaps with or without PRP. The ASC and PRP combination appears to reduce the osteonecrosis frequency and augment bone turnover and osteoclast count. The addition of BMP-2 can further improve these results (Barba-Recreo et al., 2015). Kaibuchi et al. investigated the contribution of MSC sheet transplantation in a BRONJ-like rat model (Kaibuchi et al., 2016). The MSC sheet group displayed more vessels and a higher wound healing rate than the control group and MSC intravenous injection group. Kuroshima et al. observed that noncultured SVF systemic transplantation on osteonecrosis of jaw-like lesions in mice fosters osseous and soft tissue healing of tooth extraction sockets (Kuroshima et al., 2018). Cell therapy significantly increased blood vessels and the M2/M1 macrophage ratio. SVF transplantation reduced the increase in tartrate-resistant acid phosphatase–positive (TRAP^+^) mononuclear cells (MNC) and nonattached osteoclasts from the bone surface. Kaibuchi et al. applied AT-MSC cell sheets in a MRONJ high-risk dog model (Kaibuchi et al., 2019). Mucosal wound healing was complete on the MSC sheet transplant side. Signs of inflammation and infection were observed in the control group. Histological analysis highlighted sequestrums and bacterial colonies, and immunohistological analysis showed cathepsin K-positive multinuclear cells detached from jaw bone surfaces in the control sides. Rodriguez-Lozano et al. observed similar results after BM-MSC application after tooth extraction in an animal model (Rodríguez-Lozano et al., 2020). No clinical or histological signs of MRONJ were noticed. However, the control group, without BM-MSC, exhibited MRONJ in 33% of cases. Kuroshima et al. *also* observed that transplantation of a small quantity of peripheral blood MNC (PBMNC) decreased BRONJ-like lesions marked by enhanced soft and hard tissue healing, increased M1 and M2 macrophages and promoted neovascularization (Kuroshima et al., 2019). Moreover, it reduced inflammation through polymorphonuclear cells and TNF-α expression reduction.

Twelve successful cases of cell therapy for MRONJ have been documented in the literature before the current study (Cella et al., 2011; Gonzálvez-García et al., 2013; Voss et al., 2017; De Santis et al., 2020; Bouland et al., 2021a). These studies primarily consisted of case reports, with one exception that was a case series comprising six patients. Most of these studies were performed in a one-step procedure (Cella et al., 2011; Gonzálvez-García et al., 2013; Voss et al., 2017; Bouland et al., 2021a), similar to the current approach. De Santis et al. were the only ones to perform a two-step procedure: harvesting, *ex vivo* expansion, and subsequent reimplantation into the cavity (De Santis et al., 2020). The cell sources used in these therapies were BM and AT. BMSC, BM-MSC or AT-SVF were injected with or without other adjuvant treatments after necrosectomy or bone debridement. The different associated therapeutic elements for cell injection were spongostan (Cella et al., 2011), PRP, beta tricalcium phosphate (beta-TCP), demineralized bone matrix (DBM) (Gonzálvez-García et al., 2013), autologous thrombin, and a collagen membrane (Voss et al., 2017). Out of the patients treated in these previous studies, four were undergoing antiresorptive treatment for oncological conditions, while eight were being treated for osteoporosis. In the current study, all patients had underlying oncological disorders. One patient was classified as stage I (Voss et al., 2017), nine patients were classified as stage II (Gonzálvez-García et al., 2013; Voss et al., 2017; De Santis et al., 2020; Bouland et al., 2021a), and two patients were classified as stage III (Cella et al., 2011; Bouland et al., 2021a). Soft tissue healing was observed in all cases (Cella et al., 2011; Gonzálvez-García et al., 2013; Voss et al., 2017; De Santis et al., 2020; Bouland et al., 2021a), and bone healing or regeneration was highlighted through 3D imaging in four cases (Cella et al., 2011; Gonzálvez-García et al., 2013; Bouland et al., 2021a). No recurrence was reported during follow-up.

Bone formation requires several elements: a cell source, a scaffold, tissue-inducing factors (signalling factors), and mechanical stimulation (Perez et al., 2018). Different MSC sources exist (Choi, 2009; Moreira et al., 2020). AT presents several advantages: abundance, ready availability with little discomfort, a high yield in the number of cells per unit and ethics committee agreements considering that AT is usually considered as a waste (Choi, 2009; Moreira et al., 2020). Furthermore, the BM is invaded in some malignancies, such as MM (Espagnolle et al., 2020). Consequently, BM-MSC display functional abnormalities such as IL-1 and DKK1 overexpression and early senescence (André et al., 2015). However, these functional abnormalities are not highlighted in AT-MSC (Lin et al., 2014). Different strategies have been undertaken to introduce the vasculature into tissue-engineered constructs, including scaffolds, growth factors, *in vitro* or *in vivo* graft prevascularization and coculture (Liu et al., 2015). The MSC and EPC coculture enhances both angiogenesis and osteogenesis (Sun et al., 2016). The AT-SVF, cell source we used in the current study, contains these two cell populations and is considered safe to use in clinics (Ferreira et al., 2023). In 2013, Jurgens et al. showed in an animal model that the osteogenic potential of uncultured SVF was superior to the osteogenic potential of AT-MSC (Jurgens et al., 2013). EPC foster both angiogenesis and osteogenesis via trophic factor secretion (Jia et al., 2019). EPC do not have a role in osteogenic differentiation but rather in osteoblastogenesis through angiogenesis, increasing bone formation and neovascularisation (Usami et al., 2009). Furthermore, EPC exert a dynamic role in maintaining MSC stemness and pluripotency capacities by indirect cell‒cell interactions (Wen et al., 2016). Furthermore, it has been suggested that MSC might exert their therapeutic effects mainly through secreted extracellular factors such as extracellular vesicles (Draguet et al., 2023a). MSC functions have been attributed to MSC-derived EVs. Furthermore, EV therapy displays advantages over cell therapy (Salybekov et al., 2021): 1) the cargo delivery of favourable miRNAs responsible for promoting angiogenesis, fibrosis, and cell proliferation; 2) the potential for “off-the-shelf” availability and for repetitive transplantation; 3) cell-free biological products that may be utilized as drug carrier systems in the pharmaceutical industry; and 4) reduced immunogenicity, allowing allogeneic transplantation. Several roles have been attributed to EVs, such as signalosomes, biomarkers, and therapeutic agents (El Andaloussi et al., 2023). Furthermore, EVs have emerged as a novel drug delivery vehicle (Draguet et al., 2023b). Dong et al. and Zheng et al. recently evaluated the potential contribution of EVs in MRONJ (Dong et al., 2021; Zheng et al., 2023). Zheng et al. observed that the addition of EVs fosters soft tissue and bone healing after tooth extraction in an MRONJ-like mouse model, thereby preventing MRONJ (Zheng et al., 2023). Dong et al. reviewed the following potential impacts of AT-MSC-derived EVs: promoting angiogenesis, enhancing bone remodelling and accelerating wound healing (Dong et al., 2021).

## 5 Conclusion

The potential of autologous AT-SVF within an L-PRF scaffold as a novel therapeutic approach for MRONJ was explored in this prospective study. This study involved nine patients with a total of ten MRONJ lesions, characterized by a several oncological conditions. The results were promising, with complete healing of the oral mucosa observed in 90% of the cases. No adverse reactions were observed, demonstrating the safety of the AT-SVF and L-PRF protocols. Cell analysis confirmed a heterogeneous cell population presence within the SVF, comprising EPC and MSC both playing a significant role in tissue regeneration and repair. The follow-up period, ranging from 6 to 37 months (average: 15.4 months), demonstrated no MRONJ recurrence. 3D medical imaging performed 6 months after the procedure revealed bone healing, providing further evidence of the potential efficacy of this treatment approach.

In conclusion, the current study introduces a promising therapeutic strategy for MRONJ using autologous AT-SVF within an L-PRF scaffold. While the results are encouraging, further investigation involving a larger patient cohort is warranted to understand the underlying mechanisms and to establish the long-term efficacy and safety of this innovative approach. The MRONJ complex nature in patients with oncological affections requires ongoing research and tailored management strategies.

## Data Availability

The raw data supporting the conclusion of this article will be made available by the authors, without undue reservation.
